# Coupling Phosphate-Solubilizing Bacteria with Phosphorus Supplements Improve Maize Phosphorus Acquisition and Growth under Lime Induced Salinity Stress

**DOI:** 10.3390/plants9070900

**Published:** 2020-07-16

**Authors:** Muhammad Adnan, Shah Fahad, Muhammad Zamin, Shahen Shah, Ishaq Ahmad Mian, Subhan Danish, Muhammad Zafar-ul-Hye, Martin Leonardo Battaglia, Raja Mohib Muazzam Naz, Beena Saeed, Shah Saud, Imran Ahmad, Zhen Yue, Martin Brtnicky, Jiri Holatko, Rahul Datta

**Affiliations:** 1 Department of Agriculture, The University of Swabi, Swabi 23561, Pakistan; zaminhort@uoswabi.edu.pk (M.Z.); drbeenasaeed@uoswabi.edu.pk (B.S.); 2 College of Plant Science and Technology, Huazhong Agricultural University, Wuhan 430000, China; 3 Department of Agronomy, The University of Agriculture, Peshawar 25000, Pakistan; shahenshah@aup.edu.pk; 4 Department of Soil and Environmental Sciences, The University of Agriculture, Peshawar 25000, Pakistan; ishaqmian@aup.edu.pk; 5 Department of Soil Science, Faculty of Agricultural Sciences and Technology, Bahauddin Zakariya University, Multan 60800, Punjab, Pakistan; zafarulhye@bzu.edu.pk; 6 Nutrient Management Spear Program (NMSP), Cornell University, Ithaca, NY 10001, USA; mlb487@cornell.edu; 7 Department of Horticulture, Faculty of Agriculture, University of the Poonch Rawalakot, AJ&K Rawalakot 12350, Pakistan; mohib@webmail.hzau.edu.cn; 8 Department of Horticulture, Northeast Agriculture University, Harbin 150000, China; saudhort@gmail.com; 9 Department of Horticulture, The University of Agriculture, Peshawar 25000, Pakistan; imranahmad@aup.edu.pk; 10 College of Life Science, Liniyi University, Liniyi 276000, China; 11 Department of Agrochemistry, Soil Science, Microbiology and Plant Nutrition, Faculty of AgriSciences, Mendel University in Brno, Zemedelska1, 61300 Brno, Czech Republic; martin.brtnicky@mendelu.cz; 12 Institute of Chemistry and Technology of Environmental Protection, Faculty of Chemistry, Brno University of Technology, Purkynova 118, 62100 Brno, Czech Republic; 13 Department of Geology and Pedology, Faculty of Forestry and Wood Technology, Mendel University in Brno, Zemedelska 3, 61300 Brno, Czech Republic; jiri.holatko@centrum.cz

**Keywords:** global warming, salt stress, maize, organic manures, phosphate-solubilizing bacteria

## Abstract

Global warming promotes soil calcification and salinization processes. As a result, soil phosphorus (P) is becoming deficient in arid and semiarid areas throughout the world. In this pot study, we evaluated the potential of phosphate-solubilizing bacteria (PSB) for enhancing the growth and P uptake in maize under varying levels of lime (4.8%, 10%, 15% and 20%) and additional P supplements (farmyard manure, poultry manure, single super phosphate and rock phosphate) added at the rate of 45 mg P_2_O_5_ kg^−1^. Inoculation and application of P as organic manures (Poultry and farm yard manures) improved maize growth and P uptake compared to the control and soils with P applied from mineral sources. Liming adversely affected crop growth, but the use of PSB and organic manure significantly neutralized this harmful effect. Mineral P sources combined with PSB were as effective as the organic sources alone. Furthermore, while single supper phosphate showed better results than Rock phosphate, the latter performed comparably upon PSB inoculation. Thus, PSB plus P application as organic manures is an eco-friendly option to improve crop growth and P nutrition in a calcareous soil under changing climate.

## 1. Introduction

Climate is one of the vital factors influencing soil-forming processes and properties. Although the global climate has been constantly changing throughout geological earth history, the extent to which current changes occur at a human life scale is dramatic. The global average temperature is estimated to increase by another 2–3 °C by the end of 21st century [1]. However, the impact of these changes on soil is not predictably directional, resulting in changes that may vary in strength, occurrence (i.e., permanent or periodical) and outcome (i.e., favorable or unfavorable effects). Increasing atmospheric CO_2_ concentration, temperature, drought stress, uneven precipitation and atmospheric N_2_ deposition have significant negative impact on soil functions [2]. Moreover, there is evidence that crop yields have significantly decreased due to increased salinization and calcification with increasing aridity under changing climate [3].

Phosphorus (P) is the major growth hampering mineral nutrient next to nitrogen (N) across the globe [4]. In comparison with N, P cannot be fixed by microbes from the atmosphere [5]. Approximately on 30%–40% of the land under cultivation, P deficiency is responsible for poor soil productivity [6]. In the soil, the available phosphate anions (H_2_PO_4_**^−^**^1^, HPO_4_**^−^**^2^ and PO_4_**^−^**^3^) are either adsorbed by clay surfaces [7] or form insoluble complexes like CaP and MgP with cations in alkaline soils or FeP and AlP in acidic soils [8], thus become unavailable to the plant.

Calcareous soils, typically found in the inceptisols, entisols, alfisols and vertisols soil orders, may also fix a substantial amount of P [9]. Consequently, the bioavailable in such soils is usually less than 0.1 mg kg^−1^ [10]; therefore, to attain the P requirements of a high yielding crop, regular supplementation through the exogenous application of different fertilizers is required [11]. Moreover, when removal of plant biomass for bioenergy purposes is pursued, fertilizer application rates can be substantially higher [12,13,14] and more expensive [15] compared to the grain production systems. To fulfill global P demands, chemical P fertilizers are produced at a cost of four billion USD per year [16]. However, the P use efficiency of mineral fertilizers is only around 10%–25% throughout the world [17], because significant quantities of the P in fertilizers enter into immobile pools in the soil through precipitation reactions [18]. These problems not only increase production costs, but also pollute the environment [19], which results in the need of more frequent and elaborated remediation programs to reduce the risk of P runoff to impaired water bodies [20,21]. Rock phosphate (RP), which contains 15%–20% P, is a natural, economic and readily available potential alternative to chemical P fertilizers but is not available for plant use in alkaline soils. Additionally, organic manures added to calcareous soils may form Ca bound organic P complexes, like Ca-phytates, which also cause P deficiency [22,23,24,25,26]. All these issues have compelled scientists to search for environmentally and economically feasible methods to increase crop yield and P availability using chemical, natural and manure fertilizers in P deficient soils [27].

According to an estimate by Khan et al. [28] if the reserved P in cultivated land is made bioavailable, there will be no need for additional P supplements for almost 100 years. The use of PSB in the soil is an environmentally friendly alternative to the use of mineral P fertilizers. In the soil, PSB secrete phenolic compounds, protons [29] and organic [30] and mineral acids [31] resulting in soil acidification [32] and subsequent P release from Ca_3_(PO_4_)_2_ in calcareous soils. The organic acids chelate cations, like Ca^2+^, Al^3+^ and Fe^3+^ and may increase the bioavailable P [33]. Phosphate-solubilizing bacteria my improve P availability and crop growth by promoting biologic N fixation [34], by releasing growth promoters such as IAA [35], gibberellins and cytokinins [36]. Alkaline phosphatases [37], H^+^ protonation [34,35] anion exchange, chelation and production of siderophores, hydroxyl ions, and CO_2_ may also add to improved soil and plant P nutrition [38,39,40]. Additionally, PSB inoculation has improved the yield and P nutrition of crops such as rice [41], maize [42] and other cereals [43]. Thus, phosphate-solubilizing bacteria can be efficiently used as environmentally friendly and economically beneficial substitutes for expensive P fertilizers.

Worldwide reduction in cultivable land by urbanization and industrialization is leading to a food crisis [44]. Food and Agriculture Organization [45] estimated that, by 2050, feeding a world population of 9.1 billion would require approximately 70% more food than available at present. Thus, for ensuring food security, there is a need for advanced technologies, modern cultural practices and more productive cultivars [44]. Under such scenario, phosphate-solubilizing bacteria could be utilized as an effective and economic alternative to expensive synthetic P fertilizers with a documented potential to improve crop yields and soil properties. The potential benefits of PSB, however, are not completely understood owing to their inconsistent performance in varying soil and climatic conditions [27]. We assume that PSB and P application as organic manures may nullify the ill effects of lime over growth and P uptake in maize. Thus, this study was executed to explore the role of PSB in improving maize growth and P availability from different P sources (organic, natural and chemical) in soil with varying lime levels.

## 2. Materials and Methods

### 2.1. Soil Description

A noncalcareous soil containing 4.8% lime (Gulyana soil series) was taken at the 0 to 20-cm soil depth in a field under wheat–maize rotation at the Agricultural Research Station (ARS) Baja Bam Khel (34°6’0N 72°32’0E), Distract Swabi, Khyber Pakhtunkhwa, Pakistan. The soil was a silty loam, alkaline (pH = 7.6) and non-saline (EC = 0.74 dS m^−1^) in nature, with low organic matter (0.8%) content and was deficient in total nitrogen (N = 0.08%), Olsen P (5.3 mg kg^−1^) and potassium (K = 78 mg kg^−1^) [46,47,48].

### 2.2. Material Used

The farmyard manure (FYM) and poultry manure (PM) were collected from the dairy and poultry farms of the University of Agriculture Peshawar, respectively. They were air-dried, screened, sieved (2 mm) and studied for their NPK concentration as prescribed in Table 1. The well ground RP containing 17% P was purchased from the Nuclear Institute for Food and Agriculture (NIFA), Peshawar (Table 1). The powder lime was purchased from a local market. The peat based maize PSB biofertilizer used in this study was obtained from National Agricultural Research Center (NARC) Islamabad. The inocula was examined for bacterial population and composition using Bergey’s manual of systematic bacteriology [45] on Pikovskaya’s agar media with Ca_3_(PO_4_)_2_ as insoluble P [49]. It was also analyzed for plant growth-promoting rhizobacterial (PGPR) characteristics like phosphate solubilization [50], alkaline phosphatase activity [51], siderophores [52] and indole acetic acid (IAA) [53] production and bacterial population.

### 2.3. PGPR Characteristics, Population and Composition of Applied PSB

The bacterial population in peat based maize PSB biofertilizer used in this study was 1.5 × 10^7^ CFU of PSB g^−1^ inocula (Table 2). It was further classified into *Achromobacter* (6.6%), *Agrobacterium* (3.9%), *Bacillus* (12.2%), *Burkholderia* (11.5%), *Erwinia* (10.1%), *Flavobacterium* (2.9%), *Micrococcus* (5.8%), *Pseudomonas* (15.3%), *Rhizobia* (16.8%), while 15% of the species were unidentifiable (Table 2). The inocula was capable of P solubilization (6.7 diameter of halo in mm) and producing PGPR substances like IAA (7.5 µg mL^−1^), Siderophores (6.0 diameter of halo in mm), axines (4.7 mg mL^−1^), organic acids (11 g L^−1^) as presented in Table 3.

### 2.4. Experimental Procedures

This pot study was conducted using a three factor completely randomized design (CRD) in triplicates. These three factors contained two kinds of inoculation (with and without PSB), four different P sources (SSP, RP, PM and FYM) and four doses of lime (4.8%, 10%, 15% and 20%), thus, comprising 32 treatments per replication. The soil was sterilized by autoclaving at 121 °C at 1.1 atm (approx. 16 lbs/in; 1.137 kg/cm) for a minimum of 20–30 min. The soil having 15% (V/M) moisture was filled into 96 pots amounting to 7 ± 0.01 kg soil (inclusive of natural/added lime) in such a way that four sets, each of 24 pots containing 7, 6.6, 6.3 and 5.9 kg of soil were amended with 0, 366, 716 and 1065 g of powdered lime one month before sowing to obtain 4.8 (control), 10%, 15% and 20% (M/M) lime content, respectively. The SSP, RP, PM and FYM were added to the pots at rates of 1.75, 0.82, 10.1 and 16.3 g, respectively for supplementing 45 mg P_2_O_5_ kg^−1^ soil, as per the combination of treatments. Inclusive of the N and K added by organic sources (Table 1), pots were also supplemented with 60 mg kg^−1^-N and 30 mg kg^−1^ K_2_O as urea and sulfate of potassium (SOP), respectively, at sowing time. SSP, urea, SOP and RP were added as solutions for uniform distribution in the soil.

Seeds of maize variety Azam were sterilized by using 90% ethanol for 3 min followed by 3.5% sodium hypochlorite for 30 min and inoculated (2 kg PSB inocula 25 kg^−1^ seeds ha^−1^) with PSB inocula containing 1.5 × 10^7^ CFU of PSB g^−1^ inocula (wet weight). A 50 g of seeds for each without and with PSB treatments were soaked for 2 h in sterilized distilled water and a 10% sugar solution, respectively. For PSB inoculation, A 50 g of sugar-soaked maize seeds were treated with 8 g of PSB inocula (at the rate of 2 kg PSB inocula 25 kg^−1^ seeds ha^−1^) according to the method used by Alagawadi and Gaur [54]. There were 2.5 × 10^5^ CFU of PSB per maize seed, determined by dilution plate techniques [55]. Inoculated and (control) seeds were sown at the rate of five seeds per pot and thinned to three plants per pot after germination. After sowing, the pots were placed in the open air and randomized periodically. Moisture content in pots was preserved at about 60% of field capacity during the experiment by adding water at alternate day. Normal cultural practices were applied throughout the experiment. The plants were harvested at harvest maturity and data were recorded on days to emergence, percent germination, root and shoot biomass, shoot/root ratio, plant P concentration and uptake and postharvest soil P concentrations.

### 2.5. Data Collection

Soil EC and pH were quantified in 1:5 soil water suspensions by the procedure of Rhoades [56] and Thomas [57], respectively. Soil N and K were determined by the Kjeldahl [58] Ryan et al. [59] procedures, respectively. The soil was also analyzed for lime [60], organic matter [61] contents and texture [62]. Soil P was determined by procedure of the Olsen NaHCO_3_ [63], while plant P was measured by an acid digestion method [64]. P uptake by the plant was taken as a product of P concentration and respective biomass from each pot.

### 2.6. Statistical Analysis

Descriptive statistics were calculated for the findings of PGPR characterizations by PSB. The replicated data of plant and postharvest soil properties were analyzed by F test (ANOVA) for three factorial CRD [65] using the statistical software Statistix 8.1. To test for significance among any two means, F test data were further subjected to least significant difference (LSD) test at *p* ≤ 0.05 level.

## 3. Results

### 3.1. Maize Growth Attributes

**Figure 1:**
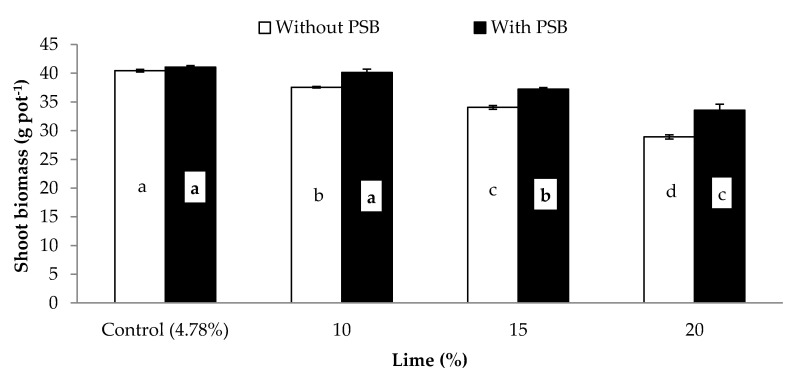
Effect of PSB on shoot biomass (g pot^−1^) of maize under varying levels of lime. Bars sharing letters are statistically comparable at *p* < 0.05 according to least significance difference (LSD) test. Error bars show standard error (n = 3).

**Figure 2:**
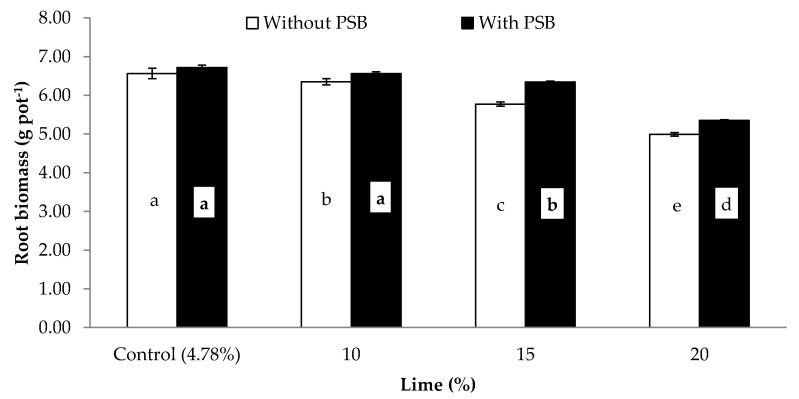
Associative effect of PSB and lime stress on root biomass (g pot^−1^) of maize. Bars sharing letters are statistically comparable at *p* < 0.05 according to least significance difference (LSD) test. Error bars show standard error (n = 3).

**Figure 3:**
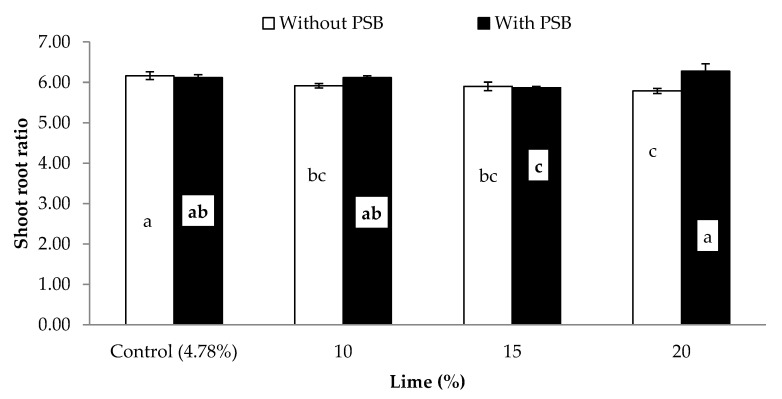
Effect of PSB on shoot root ratio of maize under varying levels of lime. Bars sharing letters are statistically comparable at *p* < 0.05 according to least significance difference (LSD) test. Error bars show standard error (n = 3).

**Figure 4:**
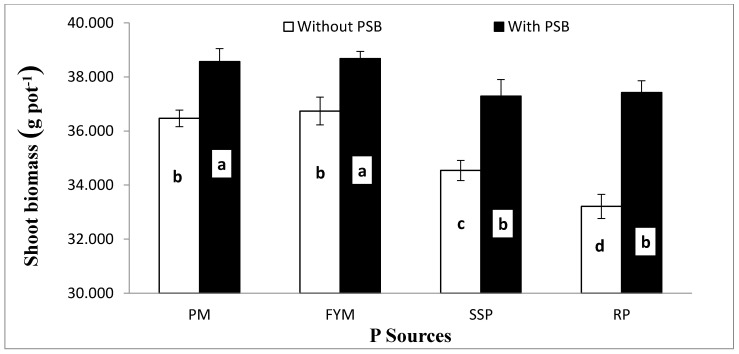
Interactive effect of PSB and P supplements on shoot biomass (g pot^−1^) of maize. Bars sharing letters are statistically comparable at *p* < 0.05 according to least significance difference (LSD) test. Error bars show standard error (n = 3).

Data concerning the influence of different P sources, liming and PSB on the germination rate (GR; %), plant height (PH; cm), shoot biomass (SB) and root biomass (RB) (both in g pot^−1^) and shoot root ratio (S/R) of maize is presented in Table 4. Except for GR, the other growth attributes were significantly affected by PSB inoculation (Table 4). Inoculation significantly increased PH, SB, RB and S/R by 5.6, 7.8, 5.5 and 2.5% respectively, when compared with the uninoculated control. Moreover, these growth attributes were also significantly affected by the different P sources. The effect of the organic sources (PM and FYM) was superior to that of the mineral P sources (SSP and RP) for all the mentioned traits. Additionally, it was observed that there were considerable intrasource differences, both in the organic (PM and FYM) and mineral (SSP and RP) sources, for the above traits. Liming adversely affected most growth attributes of maize. Except for S/R for all lime rates and germination rate for 10% lime, the other attributes showed a gradual decrease with increases in lime content compared to the control, as follows: 0%, 13% and 32% in germination rate; 6%, 11% and 21% in plant height; 4%, 12% and 23% in shoot biomass; 3%, 9% and 22% in root biomass at 10%, 15% and 20% lime content respectively. The S/R of control was similar to that of 10% and 20% liming application rates, but greater than that resulting from applying lime at the 15% content (Table 4). Analysis of variance was used to examine the responses of shoot biomass (Figure 1), root biomass (Figure 2) and shoot: root ratio (Figure 3) to the significant interaction of lime and PSB (L × PSB). Additionally, the response of SB (Figure 4) to the interaction of PSB and the P sources (PSB × PS) was also examined, as indicated in Table 4. Inoculation considerably improved both shoot and root biomass (g pot^−1^) between 3% and 16% compared to those of the uninoculated control at different concentrations of lime, except for the control lime treatment (4.8%) where statistically insignificant variation was observed with PSB addition. Further increase in the lime content beyond 10% (i.e., 10% content with PSB was similar to control with and without inoculation) caused a decrease in both the SB and RB compared to the control. The 15% lime with PSB produced results statistically at par in terms of root and shoot biomass with the 10% lime without PSB (Figure 1 and Figure 2). Similarly, the response of SB was statistically comparable for 20% lime with PSB and the 15% lime without PSB treatment (Figure 1). In both cases, the treatment with 20% lime content addition resulted in the overall lowest SB and RB (29 and 5 g pot^−1^, respectively). The associative effect of (L × PSB) for S/R revealed that, under control, 10% and 15% lime, the PSB did not perform in a superior manner to the pots without PSB, but at 20% lime, inoculated pots produced a significantly higher S/R compared to those without PSB. Maximum S/R ratios were calculated for with and without PSB inoculation at control lime which were at par to 10% lime with PSB treatment (Figure 3). The examination of the interaction of inoculation with P sources (PSB × PS) revealed that inoculation of PBS improved shoot biomass regardless of the P source used (Figure 4). The organic sources improved shoot growth considerably compared to the mineral sources, both with and without the inoculation of PBS. The performance of PM and FYM was equivalent, with and without PSB. Similarly, SSP and RP performed at par when inoculated with PSB. In addition, shoot biomass for the SSP and RP with inoculation was smaller than that of PM and FYM without inoculation. Significant responses of SB (Figure 1), RB (Figure 2) and S/R (Figure 3) to the interaction of lime and inocula (L × PSB) and SB (Figure 4) to the inocula and P sources (PSB × PS) suggested that seed inoculation with PSB can promote plant growth both in calcareous and noncalcareous soils. This addition, however, is much more crucial in calcareous soils and when mineral sources of P are utilized.

### 3.2. Maize P Concentration and Uptake

**Figure 5:**
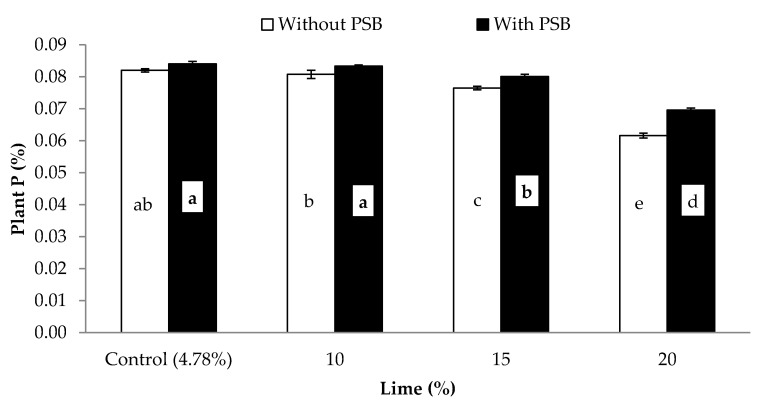
Associative effect of PSB and lime on maize P concentration (%).Bars sharing letters are statistically comparable at *p* < 0.05 according to least significance difference (LSD) test. Error bars show standard error (n = 3).

**Figure 6:**
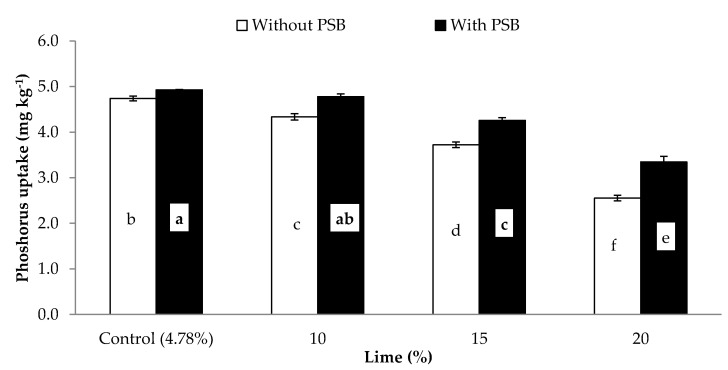
Maize P uptake (mg kg^−1^) in response to the integration of lime and PSB on of maize. Bars sharing letters are statistically comparable at *p* < 0.05 according to least significance difference (LSD) test. Error bars show standard error (n = 3).

**Figure 7:**
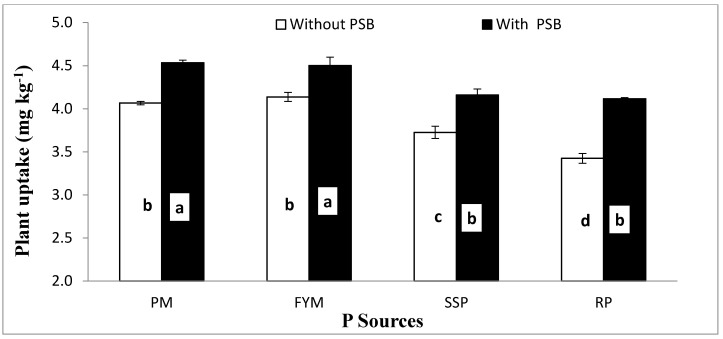
Response of maize P uptake (mg kg^−1^) to combine application of P sources and PSB. Bars sharing letters are statistically comparable at *p* < 0.05 according to least significance difference (LSD) test. Error bars represent standard error of mean for 3 values.

Data regarding the effects of P sources, liming and PSB inoculation on maize P concentration (%) and uptake (mg pot^−1^) are shown in Table 5. Inoculation significantly improved both the concentration and acquisition of P by 5.3% and 12.8%, respectively, in comparison with without PSB. The P concentration and uptake showed variable responses to different P sources. The highest P concentration (0.078%) and uptake (30.1 and 30.2 mg kg^−1^) were noted in pots amended with PM and FYM, respectively, followed by SSP in each case. The lowest P concentration (0.076%) and uptake (27.6 mg pot^−1^) were recorded for RP. With the application of lime, at any rate, the P uptake showed a decrease compared to those of the control. Applying lime at a 15% and 20% content reduced plant P concentration by 6% and 21% compared to control treatment, which was not different than lime at 10% content. A decline of 6%, 17% and 39% in the P uptake were calculated at 10%, 15% and 20% lime over control, respectively. The effect of the organic sources (PM and FYM) was superior to that of the mineral sources (SSP and RP) for both P concentration and uptake (Table 5). Both plant P concentration and uptake were significantly altered by the interaction of L and inoculation (L × PSB) (Table 5). PSB inoculation significantly improved both plant P concentration (Figure 5) P uptake (Figure 6) at all levels of lime excluding control where PSB inoculation did not have a significant effect over the control (without PSB) for plant P concentration. Liming at 15% and 20% contents significantly decreased both plant P and uptake compared to the lime control treatment, but 10% lime content did not. In addition, 15% lime + PSB had similar plant P concentration and uptake as 10% lime without inoculation. Interaction of PSB and P sources (PSB × PS) was significant for plant P uptake (Figure 7). PSB inoculation significantly improved plant P uptake over no PSB irrespective of the sources used. When comparing similar inoculation treatments, organic sources (FP and FYM) resulted in higher P uptake than mineral sources (SSP and RP). P uptake between similar inoculation treatments for PM and FYM were at par. In contrast, the effect of SSP and RP was similar when inoculated with PSB, but without inoculation, SSP increased P uptake compared to RP. Furthermore, RP and SSP with PSB had similar P uptake to that of PM and FYM sources without inoculation. These findings convene that, in alkaline soils liming is detrimental to plant P nutrition and uptake, but its damaging effect can be reduced up to 5% by application of PSB. Phosphate-solubilizing bacteria can also improve both plant P concentration and P uptake in noncalcareous soils. Based on these results it is concluded that, seed inoculation with PSB was beneficial in our study regardless of the P source utilized, and this effect is more noticeable when P is supplemented through mineral sources, especially as rock phosphate (RP). Phosphorus solubility from RP may be improved by PSB inoculation and it can be used as an environmentally friendly and economic alternative of single supper phosphate (SSP). Furthermore, P application as organic sources resulted in better results than SSP and RP application in the alkaline calcareous soils used in this study.

### 3.3. Postharvest Soil Olsen P, EC and Lime

**Figure 8:**
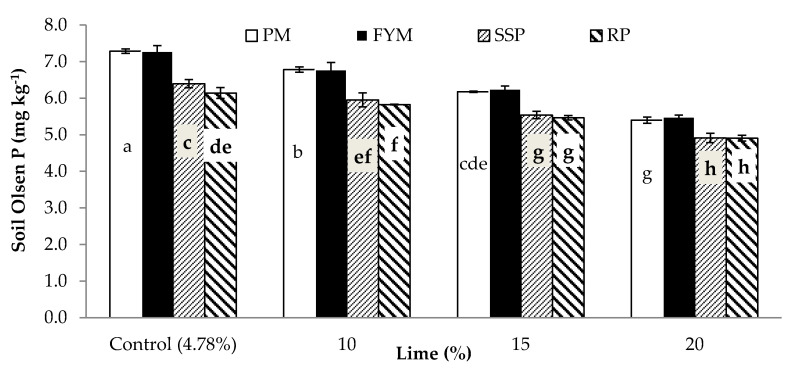
Postharvest soil Olsen P (mg kg^−1^) as affected by P sources and lime. Bars sharing letters are statistically comparable at *p* < 0.05 according to least significance difference (LSD) test. Error bars show standard error (n = 3).

The response of soil Olsen P, EC and lime content, measured following crop harvest, to inoculation, phosphorus sources and lime treatments are presented in Table 6. The inoculation treatment increased Olsen *P-*values but did not influence soil EC and lime contents. Poultry and farmyard manure increased Olsen P compared to the other two sources. Liming adversely affected Olsen P, with an increasingly detrimental effect from 10% to 20%. Addition of lime at the rate of 10%, 15% and 20% declined PSP by 77%, 14% and 24%, respectively. The influence of P supplements was at par for EC and lime. Lime and EC gradually increased with increasing content of the lime added to the soil. Liming increased postharvest soil EC by 42%, 82% and 111% and lime by 106%, 210% and 314% over control (4.8%) at 10%, 15% and 20% lime, respectively. Soil Olsen P was significantly affected by the interactive effect of lime and P sources (Figure 8). Application of lime decreased soil Olsen P irrespective of P sources. However, organic sources performed better than mineral sources at all lime contents including control (4.8%). There were no differences in soil P Olsen between PM and FYM across all lime contents. In noncalcareous soils (4.8% lime) SSP performed better than RP, whereas there were no differences in soil Olsen P between these two treatments across all other comparisons. Finally, soil having 15% lime treated with organic sources resulted in higher soil Olsen P than 10% lime + mineral sources (SSP/RP) and similar to control lime + mineral sources (SSP/RP).

## 4. Discussion

Our findings suggested that, except for germination, PSB inoculation significantly improved the rest of the growth attributes (Table 4). Our results are in line with Han et al. [66] who reported an improvement in root, shoots dry weight and yield of maize with PSB inoculation. Improvement in N, P and K uptake in pepper and cucumber has been observed with PSB inoculation [66]. Amer et al. [67] also reported an increase of approximately 120% and 97% in P uptake by *B. subtilis* and *P. fluorescens* inoculation, respectively, in common beans. We did not observe significant effect of PSB on maize germination, which contradicts the findings of Minaxi et al [68]. These authors stated that, at the germination stage, seeds obtain most of their nutrients from internal reserves, but growth hormones like auxins or gibberellins produced by PSB stimulate the process of germination. One of the possible reasons for such improvements is the PGPR behavior of PSBs, as reported herein. Our results confirm those of Sharma et al. [69] who documented that PSB enhances plant growth by more than 20 possible mechanisms, of which the most prominent are the release of valuable metabolites, such as, phytohormones, antibiotics and siderophores. Our inocula consisted of *Pseudomonas, Bacillus, Rhizobia, Burkholderia, Micrococcus, Flavobacterium, Achromobacter, Erwinia* and *Agrobacterium* (Table 2), most of which are reported as PGPR. Bashan et al. [70] and Satyaprakas et al. [71] declared *Aspergillus, Bacillus, Enterobacter, Pseudomonas, Penicillium* and *Rhizobium* as a most efficient P solubilizers. Root colonization, P solubilization, chitinase, siderophores, antibiotics, axines and ACC deaminase syntheses by PSB are the main pathways by which PSB could act as a growth promoter [72]. The PSB release phytohormones [73] and organic acids [74] which amplify P solubility and progress crop growth. PSB release substances like phosphatases [75], IAA and gibberellins [76] in addition to different organic [77] and mineral [78] acids, which ultimately improve crop growth. The PSB also increase resistance to drought and diseases [79], acidify soils [80], enhance nutrient availability [81], enhance root growth, water and nutrients uptake [82,83].

We confirmed the previous findings of Zhang et al. [84], who directly corelated P availability with soil organic matter as OM competes with P for adsorption sites. During decomposition of organic material e CO_2_ and organic acids are produced which boost the solubility of calcium Ca–P [85]. This is why P absorption in the soil is inversely related to the content of organic matter in the soil [86]. Both P concentration and uptake were improved when P was applied from organic sources (Table 5). The reason for better P concentration and uptake with organically sourced P may be due to improved soil aggregation and reduced effective surface area, which increase P mobility in the soil [87]. Messiga et al. [87] reported that organic matter may also block the CaCO_3_ surfaces and decrease the formation of Ca–P, thus, enhancing P availability and uptake by the plants.

In our study, lime induced soil salinization and calcification adversely affected crop growth (Table 4) and soil P content (Table 6). As, liming increase soil pH above 6, precipitation of P as Ca–P and micronutrient deficiencies [88]. Addition of PSB, however, may decrease the soil pH [89] by the production of organic and inorganic acids [80] and CO_2_ and the release of phosphatase enzymes [75,90,91] which enhance the availability and uptake of P by plants [92]. Inoculation significantly improved P uptake over those with no PSB irrespective of the source used (Figure 7). Based on these results it is concluded that seed inoculation with PSB is beneficial whichever P source is used, but it is crucial when P is supplemented from mineral sources, especially as RP. Phosphorus solubility from RP can be increased with PSB inoculation and it can be used as an environmentally friendly and economically beneficial alternative to SSP. Furthermore, P application from organic sources is more adventitious than SSP and RP in alkaline calcareous soils.

Additionally, the bacteria also counteract the harmful effects of liming on P nutrition. Our results are in conformity to Badr et al. [93], they also observed 58% improvement in sorghum dry matter yield in calcareous soils as a result of PSB inoculation. This was observed when PSB and RP were applied in combination rather than with the sole application of RP. Akbari et al. [94] also found improvements in available soil P and rice yield by PSB applied with RP. Sundara et al. [95] observed that PSB plus RP was more effective than sole P fertilization in sugarcane. The PSB acidify soil by producing organic [96] and mineral acids [31] like nitric and sulfuric acids [97] which enhance the solubility of P from rock phosphate.

## 5. Conclusions

PSB inoculation significantly improved maize growth, its P concentration and uptake over uninoculated (without PSB) control. The effect of organic manures (PM and FYM) was superior to that of mineral P supplements (SSP and RP) for most of the studied traits. Additionally, it was observed that liming adversely affected maize growth and P concentration and uptake and induced postharvest soil salinity and calcification. Seed inoculation with PSB was beneficial regardless of the source of P, however, the use of the bacteria was more crucial when P was supplemented from mineral sources, especially as RP. Our findings suggest that PSB inoculation may nullify the negative effects of liming on plant growth and P availability. Thus, it is suggested that P should be applied from organic sources for the improvement of crop yield and P nutrition under saline/calcareous condition. Furthermore, RP can be used as an eco-friendly and economically beneficial substitute to SSP when inoculated with PSB, otherwise, its performance is poorer than SSP in saline soils.

## Figures and Tables

**Table 1:** NPK contents of rock phosphate (RP), poultry manure (PM) and farmyard manure (FYM) used in the phosphate-solubilizing bacteria study.

Source	N	P	K
	(%)		
**RP**	–	17	–
**PM**	2.25	1.4	1.27
**FYM**	1.34	0.87	1.02

**Table 2:** Bacterial composition (%) of maize inocula used in the phosphate-solubilizing bacteria study.

Bacterial Genus	Composition (%)
*Achromobacter*	7
*Agrobacterium*	4
*Bacillus*	12
*Burkholderia*	11
*Erwinia*	10
*Flavobacterium*	3
*Micrococcus*	6
*Pseudomonas*	15
*Rhizobia*	17
Unidentified	15

**Table 3:** Plant growth-promoting rhizobacterial (PGPR) characteristics of phosphate-solubilizing bacteria (PSB) used in the present study.

PGPR Characteristics	Unit	Magnitude
Population	CFU g^−1^	1.5 × 10^7^
Auxin	mg mL^−1^	4.7 ± 0.53
IAA	µg mL^−1^	7.5 ± 0.71
P solubilization	diameter of halo in mm	7.0 ± 0.42
Siderophores	diameter of halo in mm	6 ± 0.66
Total ORGANIC ACID	g L^−1^	11 ± 0.68

**Table 4:** Maize growth as affected by phosphate-solubilizing bacteria (PSB) and soil phosphorus (P) sources in soil under varying levels of lime.

Inoculation	Germination Rate (%)	Plant Height (cm)	Shoot Biomass (g pot^−1^)	Root Biomass (g pot^−1^)	Shoot/Root Ratio
Without PSB	88.2	84.0	35.2	5.8	5.9
With PSBs	85.4	88.7	38.0	6.1	6.1
LSD (0.05)	ns	1.9	0.57	0.08	0.12
**P sources (PS)**					
SSP	84.7 c	84.5 b	35.9 b	5.9 c	5.9 bc
RP	81.9 c	81.9 b	35.3 b	5.9 bc	5.9 c
FYM	89.6 ab	88.9 a	37.7 a	6.0 ab	6.1 a
PM	91.0 a	89.0 a	37.5 a	6.1 a	6.1 ab
LSD (0.05)	5.1	2.6	0.80	0.12	0.17
**Lime (L) (%)**					
Control (4.8%)	98.6 a	95.3 a	40.4 a	6.5 a	6.1 a
10	95.1 a	89.4 b	38.8 b	6.3 b	6.0 ab
15	86.1 b	84.9 c	35.7 c	5.9 c	5.9 b
20	67.4 c	75.7 d	31.2 d	5.1 d	6.0 ab
LSD (0.05)	5.1	2.6	0.80	0.12	0.17
**Interactions**					
L × PSB	ns	ns	Figure 1	Figure 2	Figure 3
L × PS	ns	ns	ns	ns	ns
PSB × PS	ns	ns	Figure 4	ns	ns
L × PSB × PS	ns	ns	ns	ns	ns
CV (%)	10.2	5.3	3.8	3.5	4.8

Means followed by different lower letters show differences among treatments at the *p* ≤ 0.05 level. ns stands for statistically insignificant interaction.

**Table 5:** Mean comparison of main effects of inoculation, P sources and lime on postharvest plant P concentration and uptake.

Inoculation	Plant P (%)	P Uptake (mg pot^−1^)
u	0.075	26.9
With PSB	0.079	30.3
LSD (0.05)	0.0012	0.557
**P sources (PS)**		
SSP	0.08 b	27.6 b
RP	0.07 c	26.4 c
FYM	0.08 a	30.2 a
PM	0.08 a	30.1 a
LSD (0.05)	0.0016	0.787
**Lime (L) (%)**		
Control (4.8%)	0.08 a	33.8 a
10	0.08 a	31.9 b
15	0.08 b	27.9 c
20	0.07 c	20.7 d
LSD (0.05)	0.0016	0.787
**Interaction**		
L × PSB	Figure 5	Figure 6
L × PS	ns	ns
PSB × PS	ns	Figure 7
L × PSB × PS	ns	ns
CV (%)	3.71	4.78

Means followed by different lower letters show differences among treatments at the *p* ≤ 0.05. ns stands for statistically insignificant interaction.

**Table 6:** Postharvest soil Olsen P, electrical conductivity (EC) and lime content as affected by phosphate-solubilizing bacteria (PSB), phosphorus sources under varying lime.

Inoculation	Olsen P (mg kg^−1^)	Soil EC (dS m^−1^)	Total Lime (%)
Without PSB	5.9	0.97	12.3
With PSB	6.1	0.97	12.3
LSD (0.05)	0.09	ns	ns
**P Sources (PS)**			
SSP	5.7 b	0.98	12.3
RP	5.6 b	0.97	12.3
FYM	6.5 a	0.96	12.3
PM	6.4 a	0.96	12.3
LSD (0.05)	0.13	ns	ns
**Lime (L) (%)**			
Control (4.8%)	6.8 a	0.61 d	4.8 d
10	6.3 b	0.87 c	9.9 c
15	5.9 c	1.11 b	14.8 b
20	5.2 d	1.29 a	19.8 a
LSD (0.05)	0.13	0.30	0.06
**Interaction**			
L × I	ns	ns	ns
L × PS	Figure 8	ns	ns
I × PS	ns	ns	ns
L × I × PS	ns	ns	ns
Coefficient of variation (%)	3.65	5.40	0.82

Means followed by different lower letters show differences among treatments at the *p* ≤ 0.05. ns stands for statistically insignificant interaction.

## Abbreviations

Phosphorus	P
Phosphate-solubilizing bacteria	PSB
Nitrogen	N
Electrical conductivity	EC
Potassium	K
Farmyard manure	FYM
Poultry manure	PM
Single super phosphate	SSP
Rock phosphate	RP
Indole acetic acid	IAA
Plant growth-promoting rhizobacterial	PGPR
Colony farming unit	CFU
Completely randomized design	CRD
Sulfate of potassium	SOP
Germination rate	GR
Plant height	PH
Shoot biomass	SB
Root biomass	RB
Shoot root ratio	S/R
Postharvest soil P concentration	PSP
Statistically insignificant	ns
Least significance difference	LSD

## References

[1] Varallyay G. (2010). The impact of climate change on soils and on their water management. Agron. Res..

[2] Wixon D.L., Balser T.C. (2009). Complexity, climate change and soil carbon, A systems approach to microbial temperature response. Syst. Res. Behav. Sci..

[3] Lal R. (2000). Soil management in the developing countries. Soil Sci..

[4] Salimpour S., Khavazi K., Nadian H., Besharati H., Miransari M. (2010). Enhancing phosphorous availability to canola (*Brassica napus*
L.) using P solubilizing and sulfur oxidizing bacteria. Plant Biol..

[5] Ezawa T., Smith S.E., Smith F.A. (2002). P metabolism and transport in AM fungi. Plant Soil.

[6] Von-Uexkull H.R., Mutert E. (1995). Global extent, development and economic impact of acid soils. Plant Soil.

[7] Halajnia A., Haghnia G.H., Fotovat A., Khorasani R. (2009). Phosphorus fractions in calcareous soils amended with P fertilizer and cattle manure. Geoderma.

[8] Yadav H., Fatima R., Sharma A., Mathur S. (2017). Enhancement of applicability of rock phosphate in alkaline soils by organic compost. Appl. Soil Ecol..

[9] Torrent J., Barron V., Schwertmann U. (1990). Phosphate adsorption and desorption by goethites differing in crystal morphology. Soil Sci. Soc. Am. J..

[10] Chen Z., Ma S., Liu L.L. (2008). Studies on phosphorus solubilizing activity of a strain of phosphobacteria isolated from chestnut type soil in China. Bioresour. Technol..

[11] Bieleski R.L. (1973). Phosphate pools, phosphate transport and phosphate availability. Annu. Rev. Plant Physiol..

[12] Battaglia M., Fike J., Fike W., Sadeghpour A., Diatta A. (2019). *Miscanthus* ×*giganteus* biomass yield and quality in the Virginia Piedmont. Grassl. Sci..

[13] Kumar S., Lai L., Kumar P., Feliciano Y.M.V., Battaglia M.L., Hong C.O., Owens V.N., Fike J., Farris R., Galbraith J. (2019). Impacts of nitrogen rate and landscape position on soils and switchgrass root growth parameters. Agron. J..

[14] Kumar P., Lai L., Battaglia M.L., Kumar S., Owens V., Fike J., Galbraith J., Hong C.O., Faris R., Crawford R. (2019). Impacts of nitrogen fertilization rate and landscape position on select soil properties in switchgrass field at four sites in the USA. Catena.

[15] Battaglia M.L., Groover G., Thomason W.E. (2018). Harvesting and Nutrient Replacement Costs Associated with Corn Stover Removal in Virginia. Virginia Cooperative Extension Publication CSES-229NP. https://pubs.ext.vt.edu/content/dam/pubs_ext_vt_edu/CSES/cses-229/CSES-229.pdf.

[16] Goldstein A.H. (1995). Recent progress in understanding the molecular genetics and biochemistry of calcium phosphate solubilization by gram negative bacteria. Biol. Agric. Hortic..

[17] Isherwood K.F. (2000). Mineral Fertilizer Use and the Environment.

[18] Gyaneshwar P., Naresh K.G., Poole P.S.P. (2002). Role of soil microorganisms in improving P nutrition of plants. Plant Soil.

[19] Tilman D., Fargione J., Wolff B.D., Antonio C., Dobson A., Howarth R., Schindler W.H., Schlesinger D., Simberlof D., Wackhamer D. (2001). Forecasting agriculturally driven global environmental change. Science.

[20] Ketterings Q., Czymmek K. (2007). Removal of Phosphorus by Field Crops. Agronomy Fact Sheet Series. Fact Sheet #28. Nutrient Management Spear Program. Cornell University Cooperative Extension.

[21] Czymmek K., Ketterings Q., Ros M., Battaglia M., Cela S., Crittenden S., Gates D., Walter T., Latessa S., Klaiber L. (2020). The New York Phosphorus Index 2.0. Agronomy Fact Sheet Series. Fact Sheet #110. Nutrient Management Spear Program. Cornell University Cooperative Extension.

[22] Zaidi A., Khan M., Ahemad M.S., Oves M., Wani P.A., Khan M.S., Zaidi A., Musarrat J. (2009). Recent Advances in Plant Growth Promotion by Phosphate-Solubilizing Microbes. Microbial Strategies for Crop Improvement.

[23] Brtnicky M., Dokulilova T., Holatko J., Pecina V., Kintl A., Latal O., Vyhnanek T., Prichystalova J., Datta R. (2019). Long-term effects of biochar-based organic amendments on soil microbial parameters. Agronomy.

[24] Molaei A., Lakzian A., Haghnia G., Astaraei A., Rasouli-Sadaghiani M., Ceccherini M.T., Datta R. (2017). Assessment of some cultural experimental methods to study the effects of antibiotics on microbial activities in a soil: An incubation study. PLoS ONE.

[25] Molaei A., Lakzian A., Datta R., Haghnia G., Astaraei A., Rasouli-Sadaghiani M., Ceccherini M.T. (2017). Impact of chlortetracycline and sulfapyridine antibiotics on soil enzyme activities. Int. Agrophys..

[26] Meena R.S., Kumar S., Datta R., Lal R., Vijayakumar V., Brtnicky M., Sharma M.P., Yadav G.S., Jhariya M.K., Jangir C.K. (2020). Impact of Agrochemicals on Soil Microbiota and Management: A Review. Land.

[27] Khan A.A., Jilani G., Akhtar M.S., Naqvi S.M.S., Rasheed M. (2009). Phosphorus solubilizing bacteria, occurrence, mechanisms and their role in crop production. J. Agric. Biol. Sci..

[28] Illmer P., Barbato A., Schinner F. (1995). Solubilization of hardly-soluble AlPO4 with P-solubilizing microorganism. Soil Biol. Biochem..

[29] Ryan P.R., Delhaize E., Jones D.L. (2001). Function and mechanism of organic anion exudation from plant roots. Annu. Rev. Plant Biol..

[30] Chen Y.P., Rekha P.D., Arun A.B., Shen F.T., Lai W.A., Young C.C. (2006). Phosphate solubilizing bacteria from subtropical soil and their tricalcium phosphate solubilizing abilities. Appl. Soil Ecol..

[31] He Z., Zhu J. (1988). Microbial utilization and transformation of phosphate adsorbed by variable charged minerals. Soil Biol. Biochem..

[32] Jones D.L. (1998). Organic acids in the rhizosphere a critical review. Plant Soil.

[33] Kucey R.M.N. (1988). Effect of Penicillium bilajion the solubility and uptake of P and micronutrients from soil by wheat. Can. J. Soil Sci..

[34] Chaiharn M., Lumyong S. (2011). Screening and optimization of indole-3-acetic acid production and phosphate solubilization from rhizobacteria aimed at improving plant growth. Curr. Microbiol..

[35] Pathan S.I., Vetrovsky T., Giagnoni L., Datta R., Baldrian P., Nannipieri P., Renella G. (2018). Microbial expression profiles in the rhizosphere of two maize lines differing in N use efficiency. Plant Soil.

[36] Kucey R.M.N., Janzen H.H., Legett M.E. (1989). Microbially mediated increases in plant-available phosphorus. Adv. Agron..

[37] Rodriguez H., Fraga R. (1999). Phosphate solubilizing bacteria and their role in plant growth promotion. Biotechnol. Adv..

[38] Xiao Y., Wang X., Chen W., Huang Q. (2017). Isolation and identification of three potassium-solubilizing bacteria from rape rhizospheric soil and their effects on ryegrass. Geomicrobiol. J..

[39] Sugihara S., Funakawa S., Kilasara M., Kosaki T. (2010). Dynamics of microbial biomass nitrogen in relation to plant nitrogen uptake during the crop growth period in a dry tropical cropland in Tanzania. Soil Sci. Plant Nutr..

[40] Tiwari V.N., Lehri L.K., Pathak A.N. (1989). Effect of inoculating crops with phospho-microbes. Exp. Agric..

[41] Pal S.S. (1999). Interaction of an acid tolerant strain of phosphate solubilizing bacteria with a few acid tolerant crops. Plant Soil.

[42] Afzal A., Ashraf M., Asad S.A., Faroog M. (2005). Effect of phosphate solubilizing microorganism on phosphorus uptake, yield and yield traits of wheat (*Triticum aestivum*
L.) in rainfed area. Int. J. Agric. Biol..

[43] Krishnaraj P.U., Dahale S. (2014). Mineral phosphate solubilization, concepts and prospects in sustainable agriculture. Proc. Ind. Natl. Sci. Acad..

[44] FAO High-level conference on world food security, the challenges of climate change and bioenergy. Proceedings of the Soaring Food Prices, Facts, Perspectives, Impacts and Actions Required.

[45] Krieg N.R., Holt J.G. (1984). Bergey’s Manual of Systemetic Bacteriology.

[46] Danso Marfo T., Datta R., Vranová V., Ekielski A. (2019). Ecotone Dynamics and Stability from Soil Perspective: Forest-Agriculture Land Transition. Agriculture.

[47] Marfo T.D., Datta R., Pathan S.I., Vranová V. (2019). Ecotone Dynamics and Stability from Soil Scientific Point of View. Diversity.

[48] Yadav G., Datta R., Imran Pathan S., Lal R., Meena R., Babu S., Das A., Bhowmik S., Datta M., Saha P. (2017). Effects of Conservation Tillage and Nutrient Management Practices on Soil Fertility and Productivity of Rice (Oryza sativa L.)–Rice System in North Eastern Region of India. Sustainability.

[49] Gordon R.E., Haynes W.C., Pang C.N. (1973). The Genus Bacillus. Agricultural Handbook. No. 427.

[50] Nautiyal C.S. (1999). An efficient microbiological growth medium for screening phosphate solubilizing microorganisms. FEMS Microbiol. Lett..

[51] Eivazi F., Tabatabai M. (1977). Phosphatases in soils. Soil Biol. Biochem..

[52] Alexander D.B., Zuberer D.A. (1991). Use of chrome azurol S reagents to evaluate siderophore production by rhizosphere bacteria. Biol. Fertil. Soils.

[53] Vincet J.M.A. (1970). Manual for the Practical Study of the Root-Nodule Bacteria.

[54] Alagawadi A.R., Gaur A.C. (1988). Associative effect of Rhizobium and phosphate solubilizing bacteria on the yield and nutrient uptake of chickpea. Plant Soil.

[55] Holt J.G., Krieg N.R., Sneath P.H.A., Staley J.T., Williams S.T. (1994). Bergey’s Manual of Determinative Bacteriology.

[56] Rhoades J.D., Sparks D.L., Page A.L., Helmke P.A., Loeppert R.H., Soltanpour P.N., Tabatabai M.A., Johnston C.T., Sumner M.E. (1996). Salinity, electrical conductivity and total dissolved solids. Methods of Soil Analysis, Part 3, Chemical Methods.

[57] Thomas G.W. (1996). Soil pH and soil acidity. Methods of Soil Analysis, Part 3, Chemical Methods.

[58] Bremner J.M., Breitenbeck G.A. (1983). A simple method for determination of ammonium in semi-micro Kjeldahl analysis of soil and plant material using a block digestor. Commun. Soil Sci. Plant Anal..

[59] Ryan J., Estefan G., Rashid A. (2001). Soil and Plant Analysis Laboratory Manual.

[60] Loeppert R.H., Suarez D.L., Sparks D.L., Page A.L., Helmke P.A., Loeppert R.H., Soltanpour P.N., Tabatabai M.A., Johnston C.T., Sumner M.E. (1996). Carbonate and gypsum. Methods of Soil Analysis, Part 3, Chemical Methods.

[61] Nelson D.W., Sommers L.E., Page A.L. (1996). Total carbon, organic carbon, and organic matter. Methods of Soil Analysis, Part 2.

[62] Gee G.W., Bauder J.W., Klute A. (1986). Particle-size analysis. Methods of Soil Analysis. Part 1.

[63] Olsen S.R., Cole C.V., Watanabe F.S., Dean L.A. (1954). Estimation of Available Phosphorus in Soils by Extraction with Sodium Bicarbonate (No. 939).

[64] Richards L.A. (1954). Diagnosis and Improvement of Saline and Alkali Soils.

[65] Steel R.G.D., Torrie J.H. (1996). Principles and Procedures of Statistics, A Biometrical Approach.

[66] Han H.S., Lee K.D. (2005). Phosphate and potassium solubilizing bacteria effect on mineral uptake, soil availability and growth of egg plant. Res. J. Agric. Biol. Sci..

[67] Amer M.A., Abou El Seoud I.I.A., Rasmy M.R., Manar M. (2010). Biological control of *Sclerotinia sclerotiorum*, the casual agent of white basal rot disease of beans (*Phaseolus vulgaris*
L.). Alex. Sci. Exch. J..

[68] Minaxi N.L., Yadav R.C., Saxena J. (2012). Characterization of multifaceted *Bacillus* sp. RM-2 for its use as plant growth promoting bio-inoculants for crops grown in semi-arid deserts. Appl. Soil Ecol..

[69] Sharma G.D., Thakur R., Raj S., Kauraw D.L., Kulhare P.S. (2013). Impact of integrated nutrient management on yield, nutrient uptake, protein content of wheat (*Triticum aestivum*) and soil fertility in a typic Haplustert. Bioscan.

[70] Bashan Y., Kamnev A.A., de-Bashan L.E. (2013). Tricalcium phosphate is inappropriate as a universal selection factor for isolating and testing phosphate-solubilizing bacteria that enhance plant growth, a proposal for an alternative procedure. Biol. Fertil. Soils.

[71] Satyaprakash M., Nikitha T., Reddi E.U.B., Sadhana B., Vani S.S. (2017). Phosphorous and Phosphate Solubilising Bacteria and their Role in Plant Nutrition. Int. J. Curr. Microbiol. Appl. Sci..

[72] Jalili F., Khavazi K., Pazira E., Nejati A., Rahmani A.H., Rasuli S.H., Miransari M. (2009). Isolation and characterization of ACC deaminase producing fluorescent pseudomonads, to alleviate salinity stress on canola (*Brassica napus*
L.) growth. J. Plant Physiol..

[73] Islam M.T., Hossain M.M., Maheshwari D.K. (2012). Plant Probiotics in Phosphorus Nutrition in Crops, with Special Reference to Rice. Bacteria in Agrobiology, Plant Probiotics.

[74] Thakuria D., Talukdar N.C., Goswami C., Hazarika S., Boro R.C., Khan M.R. (2004). Characterization and screening of bacteria from rhizosphere of rice grown in acidic soils of Assam. Curr. Sci..

[75] Takano Y., Mori H., Kaneko T., Ishikawa Y., Marumo K., Kobayashi K. (2006). Phosphatase and microbial activity with biochemical indicators in semi-permafrost active layer sediments over the past 10,000 years. Appl. Geochem..

[76] Marschner A., Crowley D.B., Rengel Z. Interactions between rhizosphere microorganisms and plants governing iron and phosphorus availability. Proceedings of the 19th World Congress of Soil Science, Soil Solutions for a Changing World.

[77] Leyval C., Berthelin J. (1989). Interactions between *Laccacia laccata*, *Agrobacterium radiobacter* and beech roots, influence on P, K, Mg, and Fe mobilization from minerals and plant growth. Plant Soil.

[78] Zayed G. (1989). Can the encapsulation system protect the useful bacteria against their bacteriophages?. Plant Soil.

[79] Ibrahim H.I.M., Zaglol M.M.A., Hammad A.M.M. (2010). Response of Balady guava trees cultivated in sandy calcareous soil to biofertilization with phosphate dissolving bacteria and/or VAM fungi. J. Am. Sci..

[80] Wani P.A., Khan M.S., Zaidi A. (2007). Synergistic effects of the inoculation with nitrogen-fixing and phosphate-solubilizing rhizo-bacteria on the performance of field-grown chickpea. J. Plant Nutr. Soil Sci..

[81] Marschner P., White P.J., Hammond J.P., Plant Ecophysio Series (2009). The role of rhizosphere microorganisms in relation to P uptake by plants. The Ecophysiology of Plant–Phosphorus Interactions.

[82] Alexander M. (1997). Introduction to Soil Microbiology.

[83] Saghir K.M., Zaidi A., Wani P.A. (2007). Role of phosphate-solubilizing microorganisms in sustainable agriculture, a review. Agron. Sustain. Dev..

[84] Zhang B., Fang F., Guo J., Chen Y., Li Z., Guo S. (2012). Phosphorus fractions and phosphate sorption-release characteristics relevant to the soil composition of water-level-fluctuating zone of three Gorges reservoir. Ecol. Eng..

[85] Stevenson F.J. (1982). Humus Chemistry Genesis, Composition, Reactions.

[86] Hadgu F., Gebrekidan H., Kibret K., Yitaferu U. (2014). Study of phosphorus adsorption and its relationship with soil properties, analyzed with Langmuir and Freundlich models. Agric. For. Fish..

[87] Messiga A.J., Ziadi N., Morel C., Grant C., Tremblay G., Lamarre G., Parent L.E. (2012). Long term impact of tillage practices and biennial P and N fertilization on maize and soybean yields and soil P status. Field Crops Res..

[88] Fageria N.K. (1984). Response of rice cultivars to liming in Certado Soil. Pesq. Agropec. Bras. Brasilia.

[89] Adesemoye A.O., Kloepper J.W. (2009). Plant-microbes interactions in enhanced fertilizer-use efficiency. Appl. Microbiol. Biotechnol..

[90] Datta R., Kelkar A., Baraniya D., Molaei A., Moulick A., Meena R.S., Formanek P. (2017). Enzymatic degradation of lignin in soil: A review. Sustainability.

[91] Datta R., Anand S., Moulick A., Baraniya D., Pathan S.I., Rejsek K., Vranova V., Sharma M., Sharma D., Kelkar A. (2017). How enzymes are adsorbed on soil solid phase and factors limiting its activity: A Review. International agrophysics.

[92] Singh H., Reddy S. (2012). Improvement of wheat and maize crops by inoculating Aspergillus spp. in alkaline soil fertilized with rock phosphate. Arch. Agron. Soil Sci..

[93] Badr M.A., Shafei A.M., Sharaf El-Deen S.H. (2006). The dissolution of K and P-bearing minerals by silicate dissolving bacteria and their effect on sorghum growth. Res. J. Agric. Biol. Sci..

[94] Han H.S., Supanjani E., Lee K.D. (2006). Effect of co-inoculation with phosphate and potassium solubilizing bacteria on mineral uptake and growth of pepper and cucumber. Plant Soil Environ..

[95] Sundara B., Natarajan V., Hari K. (2002). Influence of phosphorus solubilizing bacteria on the changes in soil available phosphorus and sugarcane and sugar yields. Field Crops Res..

[96] Illmer P., Schinner. F. (1992). Solubilization of inorganic phosphate by microorganisms isolated from forest soil. Soil Biol. Biochem..

[97] Azam F., Memon G.H., Bashir E., Bantel R. (1996). Soil organisms. Soil Science.

